# Athlete-Specific Neural Strategies under Pressure: A fNIRS Pilot Study

**DOI:** 10.3390/ijerph17228464

**Published:** 2020-11-16

**Authors:** Inchon Park, Youngsook Kim, Seung Kyum Kim

**Affiliations:** 1 Department of Sports Science, Seoul National University of Science and Technology, Seoul 01794, Korea; pic2pac@gmail.com; 2 Department of Sports Science, Korean Institute of Sports Science, Seoul 01794, Korea; yskimosu@kspo.or.kr

**Keywords:** archery, stress, noncontact, simulation training, fNIRS

## Abstract

(1) Background: Stress and pressure during competition and training impair athletes’ performance in sports. However, the influence of mental stress on the prefrontal cortex (PFC) functioning in an athlete during the visual simulation task is unknown. The purpose of this pilot study was to investigate hemodynamic responses during the visual-simulation task that induces pressure and stress using functional near-infrared spectroscopy. (2) Methods: Ten archers and ten non-athlete collegiate students performed a visual-simulation task. Participants’ current stress levels were collected using a visual analog scale before and after the task. Average oxygenated hemoglobin (HbO), deoxygenated hemoglobin (HbR), and total hemoglobin (HbT) levels and their variability (standard deviation (SD) HbO, SD HbR, and SD HbT) were computed to compare the neural efficiency between athlete and non-athlete. (3) Results: In general, both groups exhibited increased stress levels after the simulation task, and there was no group difference in overall average hemodynamic response from PFC and dorsolateral prefrontal cortex (DLPFC). While the average hemodynamic response level did not differ between groups, variability in hemodynamic responses from the archer group showed a more stable pattern than the non-athlete group. (4) Conclusion: Under this experimental setting, decreasing the variability in hemodynamic responses during the visual simulation, potentially via stabilizing the fluctuation of PFC, was characterized by the stress-related compensatory neural strategy of elite archers.

## 1. Introduction

Studies investigating human stress responses have received extensive attention, because high levels of stress over long durations relate to the development of mental and physical health problems such as cardiovascular disorders [1,2], anxiety disorder [3], and obesity [4]. Some studies have demonstrated that moderate levels of stress are linked to an improvement in cognitive abilities [5] and physical performance [6], whereas high levels of stress are associated with an impairment of cognitive functions, particularly in cognitive flexibility [7] and executive functions [8]. Moreover, both outstanding physical abilities and cognitive skills that are susceptible to stress levels—such as perception, attention, working memory, and decision-making—are crucial for success at the highest level of sports. However, our understanding of the neurocognitive characteristics of elite athletes in sports is much less understood. In addition, the speed of information processing and movement artifacts still poses many obstacles for studying the neurological and neurophysiological processes regarding the cognitive skills in elite sports. Due to the lack of robust technologies, investigations involving high levels of cognitive processing important for sports performance have to date been restricted to the laboratory, involving responses to stimuli that are not closely related to sports performance [7,9]. Therefore, this study aimed to investigate the cortical activity during archery simulation training using functional near-infrared spectroscopy (fNIRS) to gain a better understanding of the involvement of prefrontal brain regions in stressful situations.

Brain studies associated with sports often use classic expert–novice paradigms to investigate the neurological activity of human beings with a high level of motor skills. Typically, expert–novice paradigm studies adopt two types of experimental tasks: (1) tasks unrelated to motor cognition that determine the effect of exercise training on an individual’s brain function and (2) tasks related to motor cognition, including motor execution, motor imagery, and action observation. These techniques are extremely vulnerable to motion artifacts, which poses challenges in investigating cortical activity during performance. To avoid these movements-associated issues, Del Percio and Babiloni [10] conducted a study on elite karate, elite fencing, and non-athletes, comparing the neural activity during their engaging a micro-motor control task (e.g., monopodalic upright standing). Other studies focused on brain activity regarding lower limb movement instructed participants to complete simple motor tasks such as foot or toe swinging and foot-pressing on objects [11,12]. However, these types of tasks are irrelevant to limb movements used in sports settings.

To overcome the impact of motion artifacts on the acquisition of signals, studies focusing on motor imagery or action observation have been conducted instead of motor execution tasks. Specifically, to confirm and extend the evidence for experience-dependent plasticity of cortical activity on action-observation and motor imagery, a study compared the task performance of experienced ballet dancers and non-dancers [13], while another study recruited basketball players, volleyball players, and non-athletes to complete motor imagery tasks [14]. These studies suggested that familiarity and expertise play a crucial role in cortical activity and are important factors that must be considered for task difficulty and engagement [15,16]. In addition, according to the simulation theory [17], there is a functional linkage between motor execution and the simulation of the action. Because the presence of activity in the motor system during the simulation—such as motor imagery and action observation—would place the action representation into the motor execution, the simulation theory postulates that the facilitation of the motor system during motor imagery, action observation, and motor execution are functionally equivalent [18,19].

The brain efficiency hypothesis suggests that an effective task performer exhibits a substantial decrease in brain glucose metabolic rates [20]. In particular, the reduction in neural activity and experience-dependent changes in energy usage indicates an increase in the efficiency of neural function [21]. This hypothesis has been supported by studies focused on the motor cognitive tasks of elite athletes. Previous studies revealed that the amplitude of task-related cortical areas in elite shooters [22] and professional ballet dancers [13] were lesser than the non-athlete control during the task performance. In addition, the brain efficiency hypothesis was also identified by the variability of hemodynamic responses during the tasks requiring a substantial cognitive effort. Specifically, when an individual performed a motor task with an additional cognitive load that induced mental stress, the variability of hemodynamic responses increased compared to the resting state [23,24,25]. Based on the brain efficiency hypothesis, an elite archer would have a characteristic of cortical activity under pressure and stressful situations. Because elite archers are frequently exposed to stress and pressure during training and completions, we hypothesized that the elite archer would likely show a lower level of hemodynamic responses and a more stable pattern in task-related areas (e.g., prefrontal cortex or dorsolateral prefrontal cortex) during the pressure-inducing simulation task compared to the control group. Because elite archers are frequently exposed to the pressure situation during the match or training, such experiences are expected to contribute to a reduction in cortical activity [21,22].

Regarding the continuous progress of brain imaging technology, fNIRS has attracted much attention to measure and monitor the hemodynamic responses in athletes’ cerebral cortexes. It is noninvasive and enables investigations into cortical response during task performance [26]. The experimental task in this study included a pressure-inducing situation that simulates scenarios from the actual competition. This situation also represented internal cognitive processes such as error detection, problem-solving, and motor planning for optimal outcomes. To determine the specific brain activity of elite archers, this pilot study compared the differences in prefrontal cortex (PFC) activation between elite archers and non-athlete collegiate students who participated in the archer class completing the visual-simulation task.

## 2. Materials and Methods

### 2.1. Participants

The Institutional Review Board at the Korea Institute of Sport Science approved the procedures (KISS-1905-015-01), and participants provided written informed consent before participation in the study. Ten elite archers (4 males and 6 females, M age = 26.8, SD = 4.1 years) that are currently registered in the Korea Archery Association and ten collegiate students (7 males and 3 females, M age = 23.2, SD = 3.6 years) that participated in the archery class were recruited and were right-hand dominant according to self-reports. None of the participants had a previous history of cardiovascular, pulmonary, neurological psychiatric, or other diseases that might have influenced the experimental results. All participants were asked not to consume caffeinated food or drink at least 24 h before the initiation of the experiment.

### 2.2. Experimental Equipment and Simulation Film

The simulation film used to create tension and stress for this study was recorded using a digital video camera (Sony hrx-nx30n, SONY, Minato, Japan and GoPro 4, GoPro Inc., San Mateo, CA, USA) set to show the performer’s perspective while performing under pressure. Clips were edited using Final Cut Pro software (Macromedia, Inc., San Francisco, CA, USA). Filming was conducted in the archery stadium of the national training center. To emulate the stressful and pressure situation from the performer’s perspective as closely as possible, the archer’s heartbeat as well as background sounds, including those of spectators, were included in the video clips. A panel of three specialist archery coaches selected the scenes most representative of the stressful situation and behavior of archers while shooting. The simulation film consisted of the following three scenes. (Scene 1) In the first round with the shooting time almost over, the stopwatch was highlighted to increase the participant’s pressure and tension to ensure the athlete could not shoot and set the bow down with a buzzer announcing the end of the shooting time. (Scene 2) In the third set of the tournament, the athlete is losing 0-4 and shooting despite the bow sight shaking due to pressure and tension, scoring three points by mistake. (Scene 3) A situation of a 5-5 shoot-off in which only one last shot is left to decide the winner; the archer is shooting in a situation where they can hear the sound of their heartbeat due to great pressure. All scenes were filmed using an action camera to ensure the participants could be perceived from the archer’s viewpoint, and high-speed recording was used to show the trajectory of the bow flying. Before the start of each scene, a visual cue was presented for two seconds and then short statements that describe each situation were presented for 7 s. The duration of the simulation film was 140 s.

### 2.3. Procedures

Prior to the experiment, participants were asked to rate the amount of perceived mental stress before watching the simulation film to investigate the changes in stress levels. The mental stress level was rated with a numerical rating scale with endpoints set at “no mental stress at all” (=0) and “the most intense mental stress imaginable” (=10). Participants performed another stress rating at the end of the experiment.

In the experiment, the participants were asked to rest for 30 s and were then shown a visual cue on a screen for two seconds representing the upcoming task (simulation film). During the simulation task, the athlete group was instructed to consider that they were experiencing a real match, and the collegiate group was instructed to consider that they were going through an intramural game or final test in the archery class. During the task, the participants’ hands were placed on the armrest of a chair with their hands pronated, feet close together, and bodies upright. The participants were not allowed to move their bodies or heads during the experiment.

### 2.4. Channel Configuration

**Figure 1:**
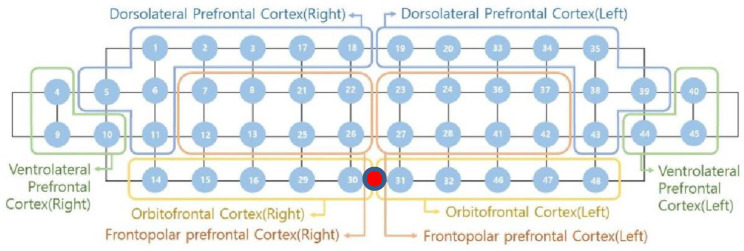
Schematic representation of the functional near-infrared spectroscopy (fNIRS) channel configuration positioned across the prefrontal cortex (PFC). PFC is divided into eight sub-area based on the Brodmann area. The red dot indicates the center of the device that located the participant’s frontopolar zone (FPz).

In this study, a near-infrared multi-channel continuous wave system (NIRSIT, OBELAB Inc., Seoul, Korea) with a sampling rate of 8.1138 Hz was employed to measure neural hemodynamic responses of the PFC via 24 emitters and 32 detectors. The device has an active detection sensor with a total capacity of 204 channels—48 of which were used in this study—covering the entire PFC area. In order to ensure that the fNIRS device is located according to the anatomical brain structure of the participants, the frontopolar zone (FPz) was used to assure comparability between all tested participants. The center of the device (Figure 1, red dot) was matched to the subject’s FPz and the bottom-most part of the device was right above the eyebrows. Based on the Brodmann area (BA), 48 channels were recorded on both sides of the dorsolateral prefrontal cortex (DLPFC, BA 9, 46), ventrolateral prefrontal cortex (VLPFC, BA 44, 45, 47), frontopolar prefrontal cortex (FPC, BA 10), and orbitofrontal cortex (OFC, BA 11). Figure 1 shows the locations of emitters and detectors with a reference point FPz (red dot) and the eight areas in this study. The wavelengths used for detecting two chromophores (oxygenated hemoglobin; HbO and deoxygenated hemoglobin; HbR) were 780 and 850 nm, respectively. For the best spatial resolution, a 3 cm distance separating the laser and detector pairs was used.

### 2.5. Data Pre-Processing

Recorded hemodynamic responses were pre-processed and analyzed using the NIRSIT analysis tool. First, the raw NIRS signal was converted into an optical intensity signal using two fourth-order Butterworth filters (low and high-pass) with cutoff frequencies of 0.5 and 0.005 Hz, respectively, to remove systemic responses caused by heartbeat and reduce slow-wave drift caused by the NIRS system [27]. The cut-off frequency of the low pass filter was sufficient to capture the fluctuation of the task-induced hemodynamics which was 20 times higher than the task period (0.025 Hz). The bad quality channels, decided by signal-to-noise ratio as <30 dB, were rejected before extraction of hemodynamics data to prevent misinterpretation and visually inspected. The optical intensity signals were transformed into the time series of HbO and HbR concentration changes using the modified Beer–Lambert law (MBLL) [28]. The concentration in hemodynamic responses (HbO, HbR, and HbT) during the experimental task is normalized to a resting baseline (−5 to 0 s) immediately preceding the onset of the task. Mean HbO levels of the baseline trials were subtracted from the mean HbO levels from the task trials to obtain normalized HbO levels from the bilateral PFC sites. The same processes were applied to HbR and HbT levels.

### 2.6. Measurements

HbO, HbR, and total hemoglobin (HbT) levels were obtained throughout the experiment. From the fNIRS data, three different types of dependent measures were obtained. To investigate the alteration pattern of HbO, HbR, and HbT levels in the PFC over the entire experimental task, the overall average value and standard deviation of HbO, HbR, and HbT were used for statistical analysis. In addition, the mean HbO, HbR, and HbT level obtained from 0 to 39 s across each scene was used to compute average HbO, HbR, and HbT values. As such, three average values (S1, S2, and S3) were present over 140 s of the experimental task. The average HbO, HbR, and HbT represented the mean hemodynamic response in each area during the experimental trials, whereas the SD HbO, HbR, and HbT represented the variability of that same signal during the simulation trials in each specific area [23]. A visual analog scale (VAS) for the current mental stress level was collected before and after the simulation task to assess the successful manipulation of mental stress levels. The VAS was used to reliably evaluate the effectiveness of the stress protocol [29].

### 2.7. Data Analysis

All dependent measures were subject to an aligned rank transformation [30] to conduct a mixed model analysis of variance (ANOVA) because of the small sample size and violation of normality assumption. A two-way mixed model ANOVA was used to determine the effect of group (athlete and non-athlete collegiate: a between-subject factor) and test (pre and post) on perceived mental stress level. A two-way mixed model ANOVA was conducted to determine the effects of group and PFC area (left and right DLPFC, VLPFC, FPC, and OFC: within-subject factor, eight levels) on the overall average and SD of HbO, HbR, and HbT values. Finally, a three-way ANOVA was conducted to determine the effects of group, scene (S1, S2, and S3: a within-subject factor, three levels), and hemisphere (left and right: a within-subject factor) on average HbO, HbR, and HbT values of the interest area (DLPFC). Statistical significance was set at *p* < 0.05. A significant main effect and interactions were examined using pairwise comparisons with Bonferroni corrections, as required. Summary data are presented as mean ± standard error (SE). The results of the experiment were visualized using Matlab (R2015b), R (v.4.0.2), and RStudio (v.1.0.136) (R and RStudio, Inc., Boston, MA, USA).

## 3. Results

**Figure 2:**
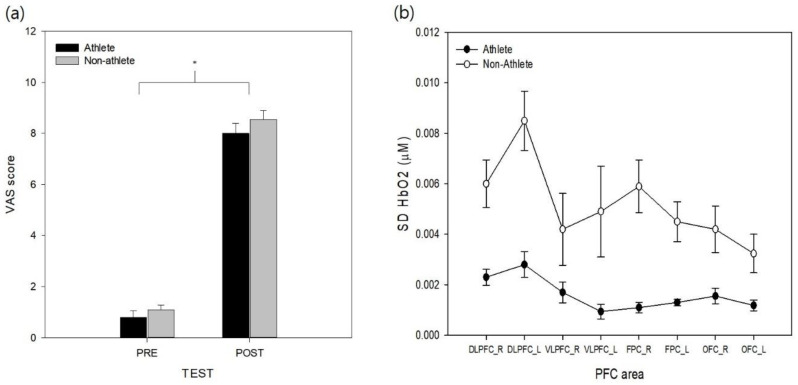
(**a**) Changes in perceived mental stress levels of athlete and non-athlete group before and after the simulation trials. (**b**) Standard deviation of oxygenated hemoglobin (HbO2) level in PFC area from athlete (●) and non-athlete (○) group during the simulation task. Error bar represents SE.

The VAS data from pre- and post-simulation tasks were analyzed to test the effectiveness of the stress manipulation. The results revealed that a significant effect of test (*F*_(1, 18)_ = 199.03, *p* < 0.001, $\eta_{p}^{2}$ = 0.97) was found on the VAS stress scores (Figure 2a). The main effect of group and its interaction with test on VAS were not found to be significant (*ps* > 0.29). The VAS values increased across the experiment, and the simulation task was found to successfully increase perceptions of stress in both athlete and non-athlete collegiate students.

To analyze the variability in cortical activity (SD for HbO) during the experimental task, SD HbO values were analyzed with two-way mixed model ANOVA (Figure 2b). The analysis revealed significant group (*F*_(1, 18)_ = 23.48, *p* < 0.01, $\eta_{p}^{2}$ = 0.55) and area effects (*F*_(7, 126)_ = 8.20, *p* < 0.001, $\eta_{p}^{2}$ = 0.19). The non-athlete collegiate group exhibited significantly higher SD HbO levels than the athlete group. Post-hoc analysis of the PFC area revealed that both sides of the DLPFC variabilities were higher than the left side of the OFC (*ps* < 0.003), and the left side of the DLPFC was the least stable area in the PFC (*ps* = 0.001–0.045). Other PFC areas were not significantly different between each other (*ps* = 0.06–0.97). The effect of group interaction with the PFC area was not found to be significant for the variation of HbO (*p* > 0.19). The analysis of SD HbR revealed significant group (*F*_(1, 18)_ = 14.73, *p* < 0.01, $\eta_{p}^{2}$ = 0.43) and area effects (*F*_(7, 126)_ = 8.67, *p* < 0.01, $\eta_{p}^{2}$ = 0.32). The non-athlete collegiate group exhibited significantly higher SD HbR levels than the athlete group (see Supplementary Figure S1a). Post-hoc analysis of the PFC area revealed that both sides of the DLPFC variabilities were higher than the left side of the OFC (*ps* < 0.01), and the left side of the DLPFC variability was higher than right OFC and left FPC (*ps* = 0.01, 0.04). Other PFC areas were not significantly different from each other (*ps* = 0.06–0.88). The interaction between the group and PFC area was not significant (*p* > 0.22). The analysis of SD HbT revealed a significant group (*F*_(1, 18)_ = 23.48, *p* < 0.01, $\eta_{p}^{2}$ = 0.45) and area effects (*F*_(7, 126)_ = 8.20, *p* < 0.01, $\eta_{p}^{2}$ = 0.31). The non-athlete collegiate group exhibited significantly higher SD HbT levels than the athlete group (Supplementary Figure S1b). Post-hoc analysis of the PFC area revealed that both sides of the DLPFC variabilities were higher than the left side of the OFC (*ps* < 0.01), and the left side of the DLPFC variability was higher than both sides of OFC and FPC (*ps* < 0.01) and left VLPFC (*p* = 0.03). Other PFC areas were not significantly different from each other (*ps* = 0.13–0.98) and the effect of group interaction with the PFC area was not significant for the HbT variability (*p* > 0.12).

**Figure 3:**
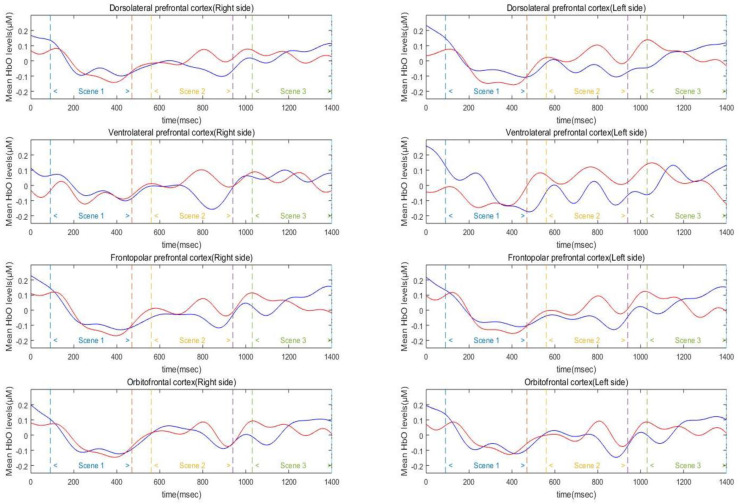
Mean HbO levels in PFC area (both sides of dorsolateral prefrontal cortex (DLPFC), ventrolateral prefrontal cortex (VLPFC), frontopolar prefrontal cortex (FPC), and orbitofrontal cortex (OFC)) of athletes (blue line) and non-athlete collegiate (red line) during the simulation task.

The overall average HbO, HbR, and HbT levels throughout the experimental trial were analyzed with two-way mixed-model ANOVA to explore the differences in mean oxygenation level in each area (Figure 3). The analysis of HbO level showed a significant main effect of the PFC area (*F*_(7, 126)_ = 2.76, *p* < 0.01, $\eta_{p}^{2}$ = 0.13). Post-hoc analysis of the main effect revealed that the cortical activity of the left VLPFC was significantly higher than that of the right VLPFC and right DLPFC (*p* < 0.05). Other cortical areas did not show a significant difference in HbO level (*p* = 0.06–0.93). The main effect of group (*p* > 0.56) and interaction between group and PFC area were not significant (*p* > 0.43). The analysis of HbR levels did not show a significant main effect of group (*p* = 0.58), PFC area (*p* = 0.21) and its interaction (*p* = 0.88, see Supplementary Figure S2a). The analysis of HbT level, also, did not show a significant main effect of group (*p* = 0.97), PFC area (*p* = 0.67) and its interaction (*p* = 0.80, Supplementary Figure S2b). 

**Figure 4:**
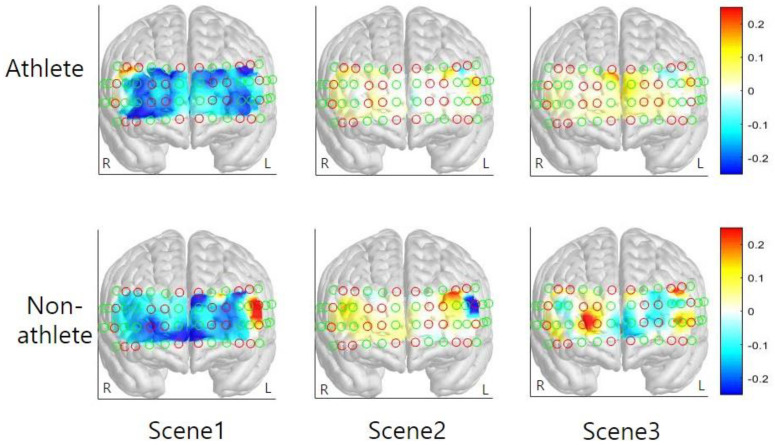
Activation maps illustrate the average HbO level obtained from 0 to 39 s across each scene for the athlete and non-athlete collegiate group. Red and green circles denote the sources and detectors, respectively. R indicates the right hemisphere and L refers to the left hemisphere.

Three-way mixed factor ANOVA was used to test for main and interaction effects of group, hemisphere, and scene on average HbO, HbR, and HbT levels in the DLPFC (Figure 4). The analysis of HbO level in the DLPFC revealed a significant main effect of scene (*F*_(2, 90)_ = 35.19, *p* < 0.01, $\eta_{p}^{2}$ = 0.89). Specifically, the DLPFC activation level of Scenes 2 and 3 was significantly higher than that of Scene 1 (*ps* < 0.001), and the difference between Scenes 2 and 3 was not significant (*p* > 0.10). The main effects of group and hemisphere were not significant (*ps* = 0.19–0.71). In addition, the effects of interaction between hemisphere, scene, and group were not found to be significant on the average HbO levels (*ps* = 0.28–0.78). The analysis of HbR level in the DLPFC revealed a significant main effect of scene (*F*_(2, 90)_ = 8.08, *p* < 0.01, $\eta_{p}^{2}$ = 0.34). Post-hoc analysis of the scene revealed that HbR level of the Scene 3 was significantly lower than the Scene 1 (*p* = 0.01). Scenes 2 and 3 were not significantly different from each other (*p* = 0.91). The main effect of group (*p* > 0.3), hemisphere (*p* > 0.2) and its interactions were not significant (*ps* > 0.14, Supplementary Figure S3a). Similar to the HbO levels, the analysis of HbT level in the DLPFC revealed a significant main effect of scene (*F*_(2, 90)_ = 12.00, *p* < 0.01, $\eta_{p}^{2}$ = 0.42). Post-hoc analysis of the scene revealed that the HbT level of Scenes 2 and 3 was significantly higher than that of Scene 1 (*ps* < 0.01) and Scenes 2 and 3 were not significantly different from each other (*p* = 0.92). The main effect of group (*p* > 0.6), hemisphere (*p* > 0.3) and its interactions were not significant (*ps* > 0.25, Supplementary Figure S3b).

## 4. Discussion

The present study compared changes in functional hemodynamic responses of the PFC between athletes and non-athletes during pressure-induced visual simulation using fNIRS. Both athletes and non-athletes showed increased mental stress levels after watching the simulation film. The overall average HbO was increased during the simulation in both groups; specifically, activation of the left VLPFC was higher than that of the right VLPFC and right DLPFC. The activation level of other cortical areas (both sides of FPC and OFC and left VLPFC and left DLPFC) were not different to each other. While group differences were not found in the average HbO, HbR, and HbT level, variability in HbO, HbR, and HbT level was found to differ between groups. The cortical activation level of the interest area, both sides of DLPFC, was increased in Scenes 2 and 3. Based on the results of this preliminary study, considerations associated with the main study are discussed in the following section.

The results of the current study associated with average HbO did not show a difference between the athlete and non-athlete groups. According to the brain efficiency hypothesis [20,22], the elite archers were expected to show a neural efficiency compared to the non-athlete. However, the findings are inconsistent with the brain efficiency hypothesis and previous studies. These results imply that both groups experienced a high level of perceived mental stress, as shown in the VAS data in this study. Although athletes are frequently exposed to extreme stress situations and competition, they also have difficulty adapting to these environments. The hemodynamic responses observed in the current study cannot be inferred for all scenarios of the simulation or performance of motor skills. The relatively economic neural activity has been observed as subjects performed a simple key pressing task [22] and a dance sequence imagery task [13]. Some discrepancies between these findings may be explained by differences in the nature of a task, neuroimaging techniques, and training schedules. For instance, stress-inducing photographs are omitted from the process of the event (i.e., injuries, critical coach, and embarrassment due to loss), possibly minimizing the magnitude of prefrontal responses [31]. In contrast, the visual-simulation film in the current investigation was mainly focused on the procedures that induce pressure. Complex and additional cognitive loads modulate the degree of prefrontal activation [32]. Furthermore, enhanced DLPFC activations during Scenes 2 and 3 suggest that these regions are crucial for error detection and online monitoring of performance [32,33]. Unlike Scene 1, participants repeatedly observed performance errors in Scenes 2 and 3. Error detection was likely to be most prominent during Scene 2, scoring 1 point by mistake, in the simulation film possibly accounting for PFC activation. The DLPFC is likely to indirectly contribute to emotion regulation through its interaction with the orbitofrontal cortex and the anterior cingulate cortex, and via these areas, with the amygdala [34]. In addition, the DLPFC is involved in emotion regulation because it plays a key role in the neural network supporting working memory function [35]. Situations such as shooting under pressure that require complex cognitive strategies: multiple sequential error detection processes and conscious control of relevant information are likely to result in profound PFC activations.

The non-athlete collegiate group exhibited significantly greater variability in hemodynamic responses in this study, suggesting that subjects exerted greater cognitive effort during the task. A previous study reported that predominantly greater HbO variability was observed in the PFC during the cognitively demanding task [24]. Subjects in the previous study showed greater HbO variability in the PFC as the reaction time and error rate increased. The authors suggested that between-group differences in neural variability might reflect systematic differences in the level of central nervous system function. In addition, consistent results regarding the variability of HbO from the current study were also reported in the postural control study that assessed the chronic ankle instability (CAI) group and healthy controls’ cortical activity in the motor cortex during single-limb stance tasks [23]. The CAI group exhibited greater HbO variability in the supplementary motor area (SMA) than the healthy control group did. The authors interpreted the results that individuals with CAI may use different corticomotor postural strategies, as indicated by the difference in variability of SMA activation. Consequently, the authors asserted that the task required substantial cognitive and motor demand from individuals with CAI, and increased variability in SMA provides evidence for an altered neural mechanism to compensate for the instability [23]. Thus, the variability of HbO values in this study indicated that the archer’s cognitive control ability is less susceptible to stress or pressure than the non-athlete counterpart.

The results of the present study’s overall average HbO demonstrate profound activation of the left VLPFC. One plausible interpretation of this result is that the subjects viewed the simulation film from the observer’s perspective rather than thinking it was what they were experiencing. The VLPFC is instrumental in empathy modulation [36,37]. The VLPFC modulates the activation of emotional systems to support emotion regulation [38]. A previous study suggested that VLPFC laterality is related to emotion processing [39]: enhancement of negative and positive emotions (up-regulations) activates the left VLPFC [40], while inhibition of these emotions (down-regulation) activates the bilateral VLPFC. For example, participants observed others experiencing negative circumstances; the observer might be eligible to empathize with the feelings of others by re-introducing the prevailing negative situation as substantially adverse. Engen and Singer [41] suggested that empathy is modulated by emotion regulation processes via a top-down manner in the VLPFC, which regulates emotional responses. Consistent with this suggestion, a previous fNIRS study reported that left VLPFC activation is directly linked to empathizing with others experiencing negative circumstances [37]. Therefore, this study’s participants may have viewed the simulation film from an observer’s perspective rather than the performer’s perspective.

## 5. Conclusions

This study is a preliminary study of research to establish an efficient simulation training environment for athletes. In this study, the pressure-induced simulation film was used to investigate the hemodynamic responses of PFC using fNIRS. The participant’s mental stress level increased following the observation of the simulation film. Archers showed a lower variability in HbO compared to non-athletes. The overall average HbO level was increased and no group difference was observed during the simulation task. Specifically, the activation level of the DLPFC was higher in Scenes 2 and 3 than in Scene 1. According to the results, the simulation film successfully manipulates the participant’s mental stress level and hemodynamic responses. In addition, specific information regarding the stress and pressure-induced situation (e.g., delayed shooting interval, swaying aiming gauge, and missed shot) was identified. While group differences in average HbO levels were not obvious, the variation in HbO from archers was more stable than that of non-athletes. In addition, the simulation film was created from the performer’s perspective as closely as possible. However, hemodynamic responses from the participants were identical to the responses from the observer’s viewpoint. To obtain more sound cortical responses as the participants perform the task in the real world, virtual reality (VR) or augmented reality (AR) technique should be considered in future studies. Furthermore, with psychological skill training combined with these techniques, additional positive effects can be expected.

The present study faces some additional limitations, which warrant a comment and which might be improved in follow-up studies. Because this was a preliminary study with a relatively small sample size, the results should be confirmed among larger samples. This study did not use a hemodynamic response function (HRF)-based general linear model (GLM) analysis. To identify a more accurate relative difference between groups or conditions, HRF-GLM analysis is recommended in subsequent studies because fNIRS cannot measure the absolute level of Hb concentration changes. Investigators may also want to explore mental stress responses using physiological factors in subsequent work. Thus, further studies should be aimed at a more diverse sample, as well as different physiological and behavioral responses to improve the generalizability of our results and outcomes.

## Supplementary Materials

The followings are available online at https://www.mdpi.com/1660-4601/17/22/8464/s1, Figure S1: Standard deviation of HbR (a) and HbT (b) level in PFC area from athlete (●) and non-athlete (○) group during the simulation task. Error bar represent SE, Figure S2: Mean HbR (a) and HbT (b) levels in PFC area (left and right DLPFC, VLPFC, FPC, and OFC) of athletes (blue line) and non-athlete collegiate (red line) during the simulation task, Figure S3: Activation maps illustrate the average HbR (a) and HbT (b) level obtained from 0 to 39s across each scene for the athlete and non-athlete collegiate group. Red and green circles denote the sources and detectors, respectively. R indicates the right-hemisphere and L refers to the left-hemisphere.
