# Supplementary material for: Athlete-Specific Neural Strategies under Pressure: A fNIRS Pilot Study
Source: Int J Environ Res Public Health. 2020 Nov 16;17(22):8464. doi: 10.3390/ijerph17228464 (PMC7697834; doi:10.3390/ijerph17228464)
Supplement: Supplementary file 1 [file ijerph-17-08464-s001.pdf]

Supplementary material

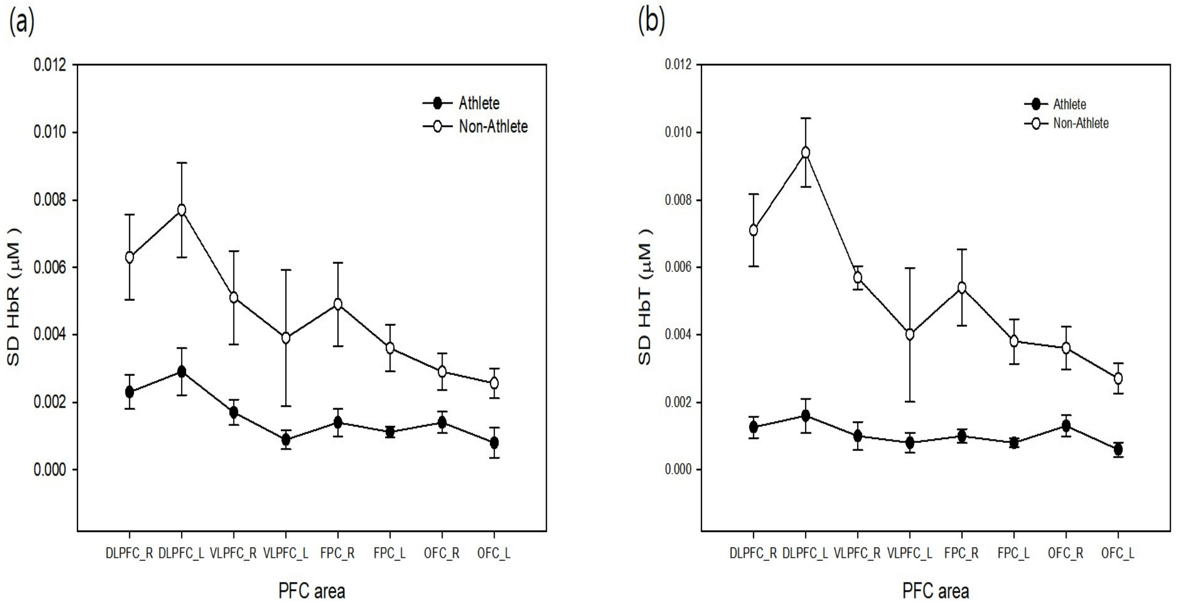

Figure S1. Standard deviation of HbR (a) and HbT (b) level in PFC area from athlete (●) and non-athlete (○) group during the simulation task. Error bar represent SE.

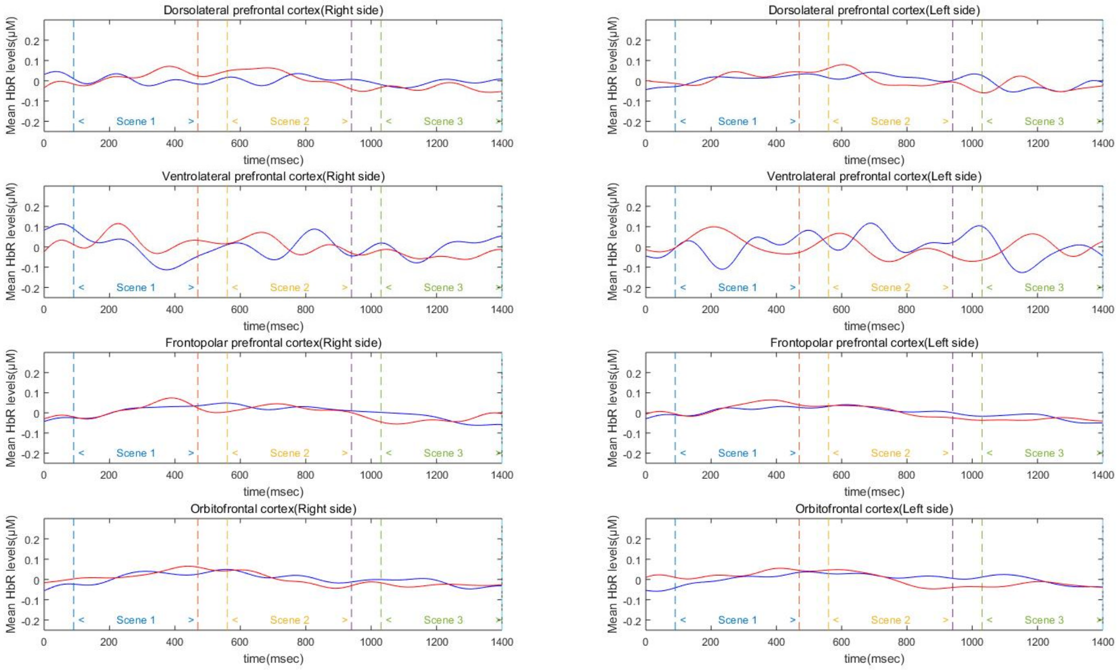

(a)

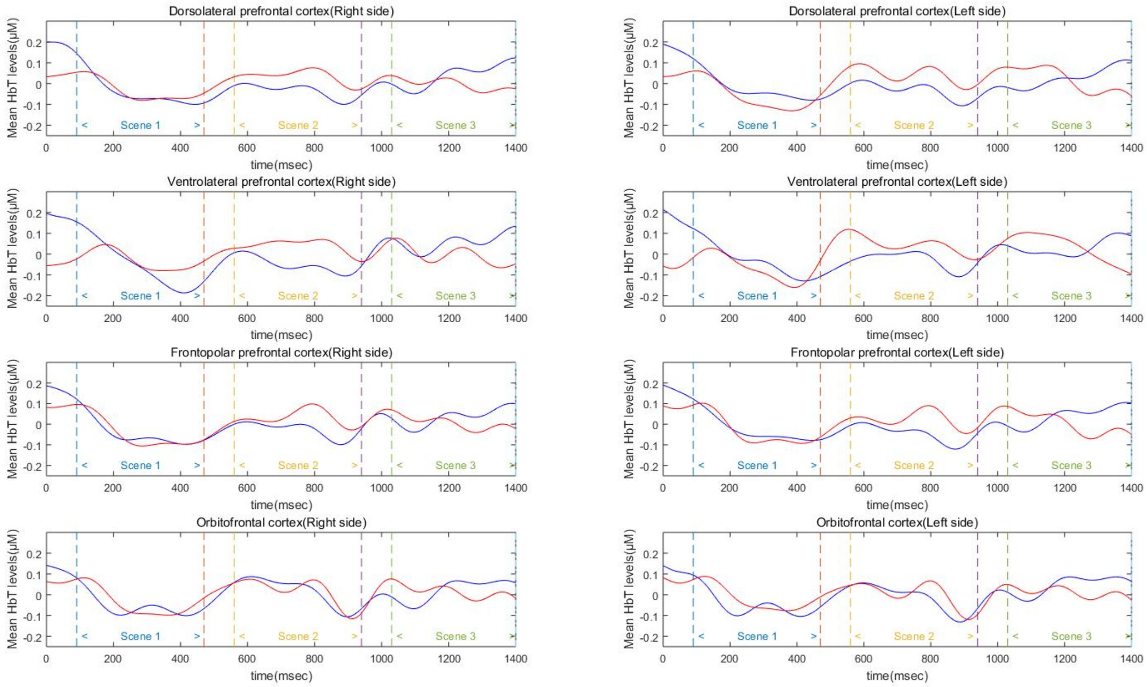

(**b**)

Figure S2. Mean HbR (a) and HbT (b) levels in PFC area (left and right DLPFC, VLPFC, FPC, and OFC) of athletes (blue line) and non-athlete collegiate (red line) during the simulation task.

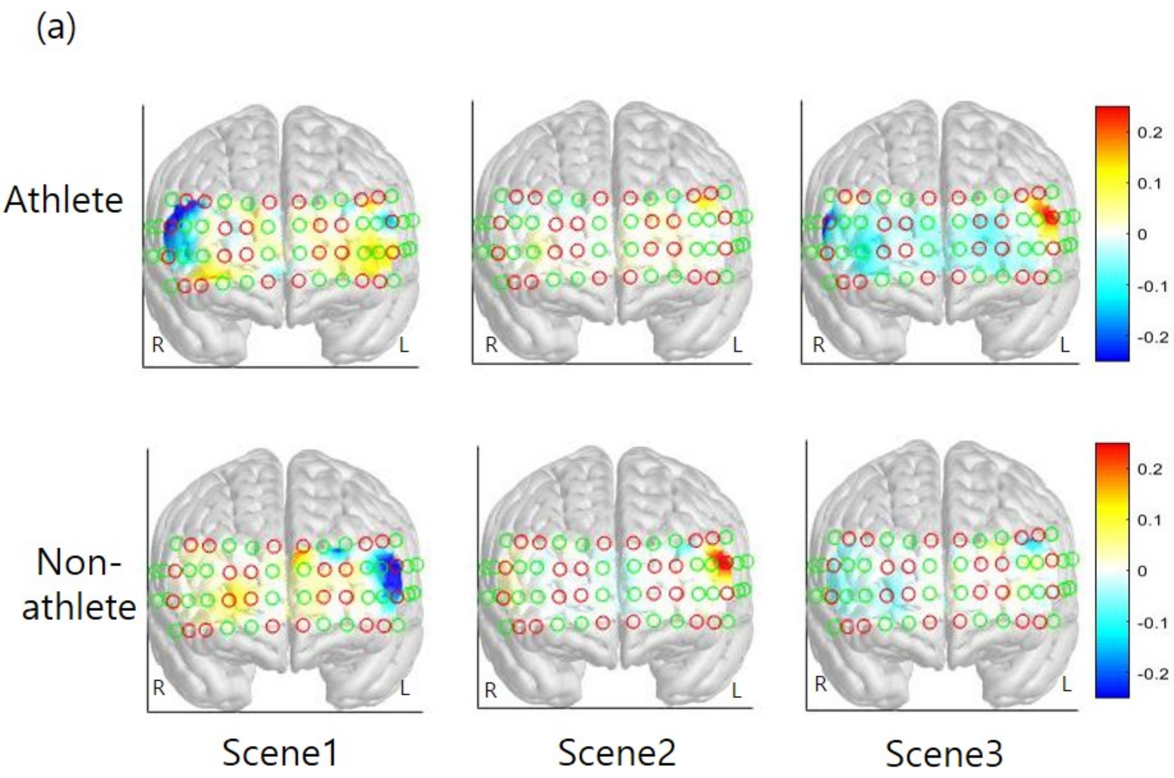

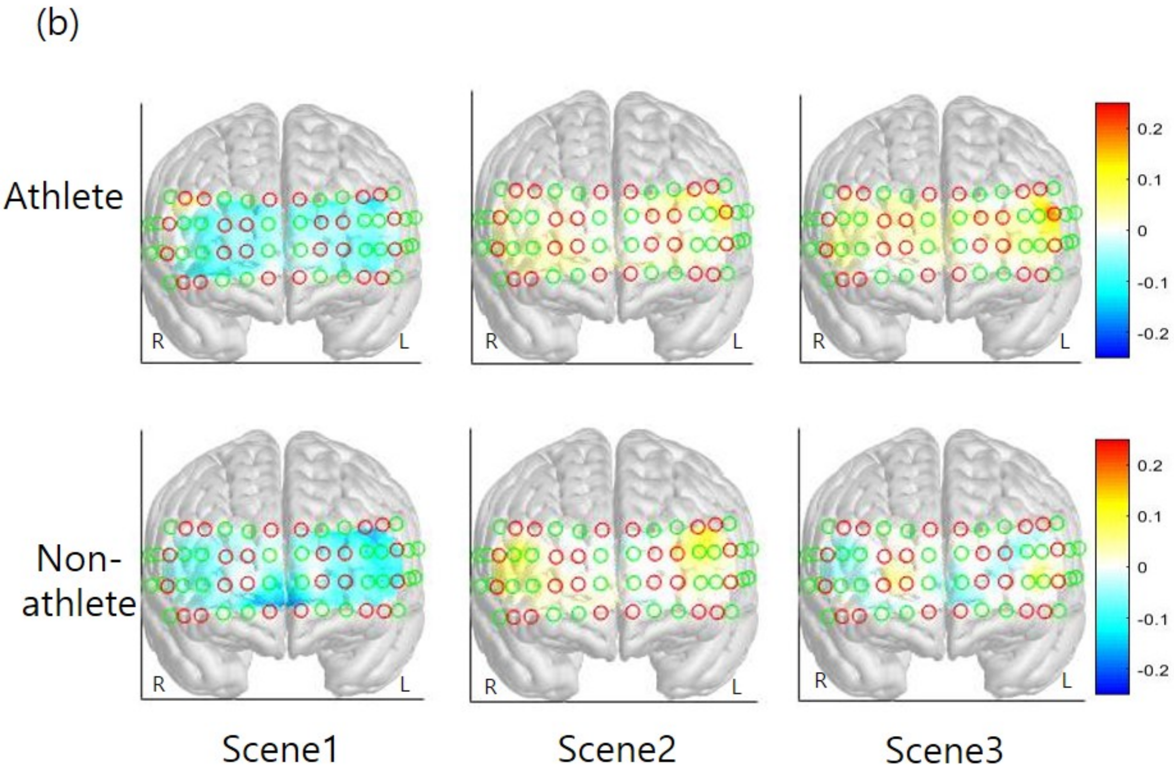

Figure S3. Activation maps illustrate the average HbR (a) and HbT (b) level obtained from 0 to 39s across each scene for the athlete and non-athlete collegiate group. Red and green circles denote the sources and detectors, respectively. R indicates the right-hemisphere and L refers to the left-hemisphere.
